# Assessment of the level of agreement in the interpretation of plain radiographs of lumbar spondylosis among clinical physiotherapists in Ghana

**DOI:** 10.1186/1471-2342-14-13

**Published:** 2014-03-29

**Authors:** Ajediran I Bello, Eric K Ofori, Oluwasegun J Alabi, David N Adjei

**Affiliations:** 1 Department of Physiotherapy, School of Allied Health Sciences, College of Health Sciences, University of Ghana, Accra, Ghana; 2 Department of Radiography, School of Allied Health Sciences, College of Health Sciences, University of Ghana, Accra, Ghana; 3 Department of Medical Laboratory Sciences, School of Allied Health Sciences, College of Health Sciences, University of Ghana, Accra, Ghan

**Keywords:** Agreement, Plain radiographs, Interpretation, Clinical physiotherapists

## Abstract

**Background:**

Objective physical assessment of patients with lumbar spondylosis involves plain film radiographs (PFR) viewing and interpretation by the radiologists. Physiotherapists also routinely assess PFR within the scope of their practice. However, studies appraising the level of agreement of physiotherapists’ PFR interpretation with radiologists are not common in Ghana.

**Method:**

Forty-one (41) physiotherapists took part in the cross-sectional survey. An assessment guide was developed from findings of the interpretation of three PFR of patients with lumbar spondylosis by a radiologist. The three PFR were selected from a pool of different radiographs based on clarity, common visible pathological features, coverage body segments and short post production period. Physiotherapists were required to view the same PFR after which they were assessed with the assessment guide according to the number of features identified correctly or incorrectly. The score range on the assessment form was 0–24, interpreted as follow: 0–8 points (low), 9–16 points (moderate) and 17–24 points (high) levels of agreement. Data were analyzed using one sample t-test and fisher’s exact test at α = 0.05.

**Results:**

The mean score of interpretation for the physiotherapists was 12.7 ± 2.6 points compared to the radiologist’s interpretation of 24 points (assessment guide). The physiotherapists’ levels were found to be significantly associated with their academic qualification (p = 0.006) and sex (p = 0.001). However, their levels of agreement were not significantly associated with their age group (p = 0.098), work settings (p = 0.171), experience (p = 0.666), preferred PFR view (p = 0.088) and continuing education (p = 0.069).

**Conclusions:**

The physiotherapists’ skills fall short of expectation for interpreting PFR of patients with lumbar spondylosis. The levels of agreement with radiologist’s interpretation have no link with year of clinial practice, age, work settings and continuing education. Thus, routine PFR viewing techniques should be made a priority in physiotherapists’ continuing professional education.

## Background

Spondylosis is a degenerative disease which can occur at any level of the spine but commonly detected at the cervical and lumbar regions [1]. It is characterized by a series of degenerative changes at the spinal end-plates, vertebral bodies and intervertebral discs with consequent formation of osteophytes and sclerosis [2]. The clinical deficits of lumbar spondylosis can range from mild to severe pain and from minimal to maximum disability with significant impact on work productivity and the quality of life of the sufferers [3,4].

Plain film radiograph (PFR) is one of the common imaging techniques used to confirm the diagnosis of lumbar spondylosis by the radiologists in most clinical settings in Ghana and it remains the mainstay in most developing African countries given its availability and cost effectiveness. Studies assessing the level of agreement of interpretation of imaging techniques among allied health professionals have been documented in literature. For instance, the outcome of meta-analysis on the degree of agreement between radiographers and radiologists to determine the radiographers’ accuracy in radiographic reports revealed accuracy level of the radiographers at 92.6% and 97.7% sensitivity and specificity respectively while nurses working at remote units also showed their respective accuracy levels at 96% for sensitivity and 87% for specificity [5,6]. Although the previous studies have assessed the interpretation of PFR among other allied health professionals, studies on agreement of physiotherapists in the interpretation of PFR are not readily available.

Based on anedoctal observation from the Ghanaian health care setting, at least two orthogonal PFR views (Anterio-posterior and lateral) are commonly examined in the preliminary assessment of patients with lumbar spondylosis by the physicians and orthopaedic specialists. Eventhough the interpretation of PFR remains within the purview of the radiologists, physiotherapists often perform this task either as extended scope of practice or as routine procedure during objective assessment. With the emergence of physiotherapy practice from under the cover of traditional referral system to self-referral methods of health care delivery particularly in wellness and musculoskeletal fields, Ghanaian physiotherapists are required to extend their horizon to be relevant in the ongoing global health reform [7]. Correct interpretation of PFR among physiotherapists becomes necessary in order to identify possible contra-indications to some physiotherapy techniques and/or modification of treatment plans accordingly to change the natural course of the presented conditions [8].

Given the growing reliance on radiographic imaging and the evolvement of first contact practice in some allied health professionals, it is necessary to appraise the skills of the relevant available health care professionals in the interpretation of imaging techniques. This study therefore determined the level of agreement in the interpretation of PFR of lumbar spondylosis between the physiotherapists and radiologists in Ghana.

## Methods

### Participants

Forty-one (41) clinical physiotherapists participated in this cross-sectional survey. They were recruited through sample of convenience from the Government hospitals and private physiotherapy clinics located at four of the ten Regions in Ghana. Clinical physiotherapists who were registered members of Ghana Physiotherapy Association and those who had practiced for at least one (1) year were included in the study. Retired physiotherapists and those in academics were excluded.

### Design of an assessment guide

Three separate PFR (two antero-posterior and one lateral views) were selected from a pool of PFR of patients undergoing physiotherapy for lumbar spondylosis at the selected hospitals and clinics. The selection was based on the clarity of the films, availability of common lumbar spondylosis pathology, coverage of peripheral body segments (bilateral pelvis and hips), and post production period not exceeding three (3) months. The selected films were shown to an experienced independent radiologist who in turn identified the pathological features and listed them as gold standard to serve as an assessment guide (Appendix 1). The listed pathological features were found to be comparable and consistent to that obtained from twenty retrospective radiologists’ reports on patients with lumbar spondylosis. A total of 24 features were identified by the radiologist from three PFR with each consisting 8 features. The assessment guide was subjected to test re-tests reliability among seven experienced radiologists whose reports were not included in the retrospective reports to scrutinize the list. A good internal consistency of 0.79 Chrombach’s alpha value was obtained through the evaluation process [9]. Correct identification of a pathological feature attracts one (1) point while wrong identification or non-identification attracts 0 point. The score range on the form is 0–24 in which the minimum obtainable score is zero (0) while the maximum obtainable score is 24. The scoring is interpreted as follow: 0–8 points (low level of agreement), 9–16 points (moderate level of agreement) and 17–24 points (high level of agreement).

### Procedure

The Ethics and Protocol Review Committee of the School of Allied Health Sciences, University of Ghana approved the proposal for this study. An information sheet containing the title and the intent of the study was made available to the participants and their written consents were obtained. A self-designed data capturing form was used to obtain demographic data such as age, sex and academic qualification and clinical data including number of years of clinical experience, work settings and the number of continuing professional development (CPD) programme attended in the last 5 years (Appendix 2). The three plain film radiographs (PFR) of lumbar spondylosis from which the assessment guide was developed were viewed by the physiotherapists in the presence of the researchers and in no particular order to avoid stereo-typed interpretation. They were required to list the identifiable patho-anatomical features observed in the PFR. A time commitment of 30 minutes was expected of the participants for the completion of the entire procedure. On completion of the interpretation and documentation process, the researchers collected the findings and evaluated each physiotherapist using the assessment guide.

### Data analysis

Data were analyzed using the SPSS version 19.0 software. Descriptive statistics of frequency, percentage, mean and standard deviation were used to summarize the data. One sample Kolmogorov-Smirnov test was explored to test the data for normalcy. Comparison of PFR interpretation between the physiotherapists and the radiologist was tested with one sample t test while Fisher’s exact test was used to determine the associations between levels of agreement and the physiotherapists’ demographic and clinical variables at alpha level of 0.05.

## Results

Forty-one (41) physiotherapists participated in this study comprising 17 (41.5%) males and 24 (58.5%) females. The demographic and clinical profiles of the participants are presented in Table 1. Most physiotherapists, 25 (61%) were within the age range 26–30 years and 36 (88%) of them hold bachelor’s degree while five (12%) had masters’ degree. The study also showed that majority of the physiotherapists 24 (58.5%) worked in teaching hospitals while 3 (7.3%) worked in general hospital. Twenty-five (61%) had been practicing for less than or a total of four (4) years as compared to 16 (39%) who had worked for 5 to 9 years. Twenty-four (58.6%) of the physiotherapists had attended less than or a total of two continuing professional development (CPD) programmes while 6 (14.6%) had attended less than or a total of 6 CPD programmes.

**Table 1:** Demographic and clinical profiles of the physiotherapists

**Variable**	**Number**	**Percentage**
**Sex**		
Male	17	41.5
Female	24	58.5
Total	41	100
**Age group (years)**		
21-25	1	2.4
26-30	25	61.0
31-35	11	26.8
36-40	2	4.9
41-45	1	2.4
46-50	1	2.4
Total	41	100.0
**Academic qualification**		
BSc.	36	87.8
MSc.	5	12.2
Total	41	100
**Years of clinical practice**		
0-4	25	61.0
5-9	16	39.0
Total	41	100.0
**Work setting**		
Regional hospital	10	24.4
Teaching hospital	24	58.5
General hospital	3	7.3
Private hospital	4	9.8
Total	41	100.0
**CPD programme attendance**		
0-2	24	58.6
3-5	11	26.8
≥6	6	14.6
Total	41	100.0
**Preference of radiograph view**		
Antero-posterior	5	12.2
Lateral	15	36.6
Neither	21	51.2
Total	41	100.0

**Key**: CPD = Continuing professional development.

Results of the categorical levels of assessment shows that 4 (9.8%) physiotherapists scored between 0 and 8 points (Low); 35 (85.4%) scored between 9 and 16 points (Moderate); 2 (4.9%) scored between 17 and 24 points (High). The levels of agreement were significantly associated with academic qualification (0.006) and the sex (p = 0.001) of the physiotherapists. However, the levels of agreement were not significantly associated with the age group (p = 0.098), work setting (p = 0.171), year of clinical practice (p = 0.666), preferred PFR views (p = 0.088) and the number of CPD attended (p = 0.069) (Table 2).

**Table 2:** **Fisher**’**s exact analysis for the associations between the physiotherapists**’ **levels of agreement of PFR interpretation and their clinical and demographic characteristics**

**Variables**	**Low n (%)**	**Moderate n (%)**	**High n (%)**	**Fisher’s exact test p value**
**Age (years)**				
21–25	0 (0.0)	1 (100)	0 (0.0)	0.098
26–30	2 (8.0)	20 (80.0)	3 (12.0)	
31–35	0 (0.0)	11 (100)	0 (0.0)	
36–40	0 (0.0)	2 (100)	0 (0.0)	
41–45	0 (0.0)	0 (0.0)	1 (100)	
46-50	0 (0.0)	1 (100)	0 (0.0)	
**Qualification**				
B.Sc	2 (5.5)	32 (88.8)	2 (5.5)	0.006*
M.Sc	0 (0.0)	3 (60.0)	2 (40.0)	
**Work setting**				
Regional hospital	1 (10.0)	8 (80.0)	1 (10.0)	0.171
Teaching hospital	1 (4.1)	21 (87.5)	2 (8.3)	
General hospital	0 (0.0)	2 (66.7)	1 (33.4)	
Private hospital	0 (0.0)	4 (100)	0 (0)	
**Years of practice**				
0–4	2 (8.0)	22 (88.0)	1 (4.0)	0.666
5-9	0 (0.0)	13 (81.2)	3 (18.7)	
**No of CPD attended**				
0–2	1 (4.2)	22 (91.7)	1 (4.2)	0.069
3–5	1 (9.0)	9 (81.8)	1 (9.0)	
≥6	0 (0.0)	4 (66.7)	2 (33.4)	
**Sex**				
Male	0 (0.0)	4 (23.5)	13 (76.4)	0.001*
Female	2 (8.4)	0 (0.0)	22 (91.7)	
**Preferred view**				
Anterio-posterior view	1 (20.0)	4 (80.0)	0 (0.0)	0.088
Lateral view	0 (0.0)	13 (86.7)	2 (13.4)	
Both	1 (4.7)	18 (85.7)	2 (9.5)	

**Key**: *Significant at p < 0.05.

Exploration of one sample Kolmogorov-Smirnov test for normalcy of data shows no significant difference in the dataset distribution (p = 0.207) thereby justifying the adoption of one sample t test analysis. The mean score of agreement (12.7 ± 2.6) for the physiotherapists was significantly lower (p = 0.001) than the gold standard point of 24 (Radiologist’s score).

## Discussion

The main focus of this study was to assess the level of agreement of interpretation of plain radiographs (PFR) between the radiologists and physiotherapists in Ghana. The mean score of interpretation of the physiotherapists compared to that of the radiologists in this study was 12.7 (approximately 50.0% of the total point) which was statistically lower than the required scores. This finding falls short of expectation giving the high agreement of other health care professionals with the radiologists in the previous studies [5,6]. For instance, Brealey et al. reported high accuracy levels for radiographers at 92.6% and 97.7% sensitivity and specificity respectively [5] while Benger submitted that nurses working in remote units had 96% sensitivity and 87% specificity in the interpretation agreement [6]. These variations might not be unconnected with the different settings in which the studies were conducted.

In addition, approximately 85.4% of the physiotherapists’ scores were within the moderate range (9–16 points) which implies that majority of the participants’ performances were within the average in PFR interpretation. Although PFR viewing is a common routine procedure in physiotherapy, it seems little priority has been placed on its techniques in the series of their continuing education programmes. Presumably, they might have acquired their rudimentary skills through personal efforts and interest. These speculations may be justifiable given the crops of young generation of the practicing physiotherapists in Ghana.

In the same vein, the levels of agreement in the interpretation of PFR by the physiotherapists’ were significantly associated with their academic qualification and sex. Most of the physiotherapists were first degree holders implying that they were still at the minimum entry level of the profession as stipulated by the World Confederation for Physical Therapy [10]. Post-graduate training programme in the field of physiotherapy is yet to take off in Ghana, thus the few physiotherapists with masters degree had to divert to other fields that may not directly impact on their clinical proficiency. The link between gender and level of agreement may be ascribed to the relative proportion of the sampled male and female physiotherapists in this study. Contrarily, the age, work settings, years of clinical practice and the preferred PFR views have no significant impact on the level of agreements. These findings suggest that the physiotherapists’ disposition towards PFR viewing rather than the selected clinical variables are the influencing factors. Further, specialty physiotherapy training and practice in Ghana are yet to be established, hence daily practice cuts across the branches of the profession without specific emphasis on orthopaedics-related cases where x-ray viewing and interpretation are mostly needed.

A shift in the focus of health care delivery from the hospital to the community, are placing increased demands on physiotherapists to embrace primary-care systems. Available evidence suggests that physiotherapists’ involvements in the primary health care setting are beneficial to patients suffering from musculoskeletal problems such as arthritis and low back pain [11]. Indeed, the convergence of rising health care costs and physician shortages have made health care transformation a priority in many developing countries resulting in the emergence of new models of care that often involve the extension of the scope of practice for most allied health professionals. Physiotherapists in extended scope roles must emerge as key providers in such new models, especially in settings providing services to patients with musculoskeletal disorders [12].

### Limitation of the study

The findings from this study are limited by the use of very few plain film radiographs of patients suffering from lumbar spondylosis with which the participants were assessed. Sampling over sufficient numbers of patients’ x-rays would have ensured external validity of the results. These observations should be the focus of the future study.

## Conclusions

We conclude that the sampled Ghanaian physiotherapists had disproportionate level of agreement in the interpretation of PFR of patients with lumbar spondylosis compared to that of the Radiologist. The sex and academic qualification of the sampled physiotherapists had significant bearing on their level of agreement. The outcomes of this study thus underscore the urgent need to include basic training in the interpretation of plain radiographs into the continuing professional development programme of physiotherapists in Ghana.

## Appendix 1

### Assessment guide

The underlisted three plain film radiographs (PFR) A, B, and C contain the common degenerative features found in patients diagnosed with lumbar spondylosis in two views (anterio-posterior and lateral):

PFR A: Lumbar spine only (anterio-posterior view)

PFR B: Lumbar spine + Pelvis (anterio-posterior view)

PFR C: Lumbar spine only (Lateral view)

The following (8) features were the pathological features found in each of the PFR.

1. Marginal osteophytes on lumbar vertebrae

2. Decreased vertebral body height

3. Decreased lumbar intervertebral disc space

4. End plate sclerosis of articular surfaces in the lumbar region

5. Scoliotic lumbar vertebrae

6. Exaggerated lumbar lordosis

7. Straightening of the lumbar spine (reduced normal lumbar lordosis)

8. Vacuum phenomenon

Mark each correctly identified pathological feature as 1 point and wrong identification or non-identification as 0 point. The total score is 24 ranging from 0–24 in which the minimum obtainable score is zero (0) while the maximum obtainable score is 24. The scoring is interpreted as follow: 0–8 scores (low level of agreement), 9–16 scores (moderate level of agreement) and 17–24 scores (high level of agreement).

## Appendix 2

### Data capturing form

**Instruction**: Kindly supply the following information as applicable to you

**Section A**: **Demographics**

1. AGE: 21–25 □ 26–30 □ 31–35 □ 36–40 □ 41–45 □ 46–50 □ 51–55 □ 55 □

2. SEX: Male □ Female □

3. ACADEMIC QUALIFICATIONS (kindly tick more than one if applicable)

Diploma □ B. Sc. □ M. Sc. □ Ph. D □

Others (specify)……………………………………………..

1. Are you a registered member of the Ghana Association of Physiotherapists?

Yes □ No □

**Section B**: **Clinical variables**

1. In which of the following work settings do you practise?

Teaching Hospital □ General Hospital □ Regional Hospital □ Private □

1. How many years of Clinical experience (excluding internship) do you have? ……………..

2. How many Orthopaedics related Continuous Professional Development (C.P.D.) programme (workshop, seminar, conference e.t.c.) have you attended in the last 5 years? ………

3. Which view of the plain Radiograph would you request for when assessing a patient suspected to have Lumbar spondylosis?

Antero-Posterior □ Lateral □

## Competing interests

The authors declare that they have no competing interests.

## Authors’ contributions

AB responsible for the design of the manuscript for intellectual presentation. He also contributed to the collection, management, analysis and interpretation of data. EO, assisted in the collection, management, analysis and interpretation of data. OA, participated in the collection of data and final editing of the manuscript. DA participated in data interpretation and analysis. All authors read and approved the final manuscript.

## Pre-publication history

The pre-publication history for this paper can be accessed here:

http://www.biomedcentral.com/1471-2342/14/13/prepub
